# 治疗前后NLR和PLR对进展期非小细胞肺癌一线化疗疗效及预后的预测价值

**DOI:** 10.3779/j.issn.1009-3419.2018.06.02

**Published:** 2018-06-20

**Authors:** 福梅 易, 阳春 顾, 森 陈, 燕娥 刘, 文琤 尹, 煜 张, 宝山 曹

**Affiliations:** 100191 北京，北京大学第三医院肿瘤化疗与放射病科 Department of Medical Oncology and Radiation Sickness, Peking University Third Hospital, Beijing 100191, China

**Keywords:** NLR, PLR, 肺肿瘤, 化疗疗效, 总生存期, NLR, PLR, Lung neoplasms, Response, Overall survival

## Abstract

**背景与目的:**

中性粒细胞淋巴细胞比值(neutrophil-to-lymphocyte ratio, NLR)和血小板淋巴细胞比值(platelet-to-lymphocyte ratio, PLR)是机体系统性炎症的体现, 与多种肿瘤的预后有关。本研究旨在探讨NLR、PLR及其动态变化对非小细胞肺癌(non-small cell lung cancer, NSCLC)一线化疗疗效和预后的影响。

**方法:**

回顾性分析68例2008年4月-2015年4月于北京大学第三医院接受一线化疗、符合入组标准的进展期NSCLC住院患者, 采集一线化疗前和2周期化疗后的NLR和PLR, 应用*Kaplan-Meier*方法进行生存分析, 单因素和*Cox*多因素分析NLR、PLR及其动态变化和各种临床特征与一线化疗疗效和总生存期(overall survival, OS)之间的关系。

**结果:**

NLR在2周期化疗后显著下降, NLR2(2周期化疗后NLR)和NLR0(治疗前NLR)分别为(2.69±2.06)和(3.94±2.12)(*P*=0.000), PLR化疗前后无显著变化(*P* > 0.05);NLR2与一线化疗2周期和4周期的疗效显著相关(*P* < 0.05), 疾病进展患者中高NLR2的比例为100.0%, 显著高于部分缓解和疾病稳定组(*P* < 0.05);NLR0、NLR2和PLR0与OS显著相关(*P* < 0.05), 但与患者年龄、体力状况、病理类型、肿瘤分期、治疗状态、治疗方案均无关(*P* > 0.05);单因素分析显示OS与NLR0、NLR2、PLR0、二线治疗状态、二线方案选择、一线化疗2周期疗效、4周期疗效显著相关(*P* < 0.05), 与肿瘤分期、三线治疗状态及放疗状态无关(*P* > 0.05);多因素分析表明NLR0(*P*=0.004)、一线化疗4周期后疗效(*P*=0.022)、二线治疗状态(*P*=0.007)是OS的独立预测因素。

**结论:**

治疗前NLR与NSCLC的预后显著相关, 化疗后NLR与一线治疗疗效显著相关。因此, NLR是预测进展期NSCLC一线化疗疗效和预后的理想指标, 也是治疗NSCLC潜在的干预靶点。

## Abstract (en)

**Background and objective:**

Neutrophil-to-lymphocyte ratio (NLR) and platelet-to-lymphocyte ratio (PLR), indexes of systemic inflammation, have been associated with worse survival for many types of cancer.The aim of this study is to investigate the impact of NLR and PLR on overall survival (OS) and to explore the value of changes in the NLR and PLR with treatment as a response indicator in non-small cell lung cancer (NSCLC).

**Methods:**

A total of 68 NSCLC patients in Peking University Third Hospital were eligible for retrospective analysis between April 2008 and April 2015.The pretreatment and posttreatment NLR and PLR in all patients were calculated based on complete blood counts.Potential prognostic factors such as age, gender, performance status, histology, stage, response to chemotherapy, NLR and PLR were analyzed.NLR and PLR were assessed at baseline and during chemotherapy treatment.OS was calculated by the *Kaplan-Meier* method.Univariate and multivariate *Cox* regression analyses were performed to determine the associations of the PLR, NLR and clinical features with OS.

**Results:**

Among the 68 cases, the values of the posttreatment NLR after two cycles of chemotherapy (NLR2) and the pretreatment NLR (NLR0) were (2.69±2.06) and (3.94±2.12), respectively.NLR2 was significantly lower than NLR0 (*P*=0.000).There was no difference between the pretreatment PLR (PLR0) and the posttreatment PLR after two cycles of chemotherapy (PLR2) (*P* < 0.05).NLR2 significantly correlated with the response of first line treatment with two or four cycles of chemotherapy.The proportion of high NLR2 in the patients with progression disease was 100.0%, significantly higher than the proportion of high NLR2 in the patients with partial response or stable disease.NLR0, PLR0 and NLR2 were significantly correlated with the OS (*P* < 0.05), but not with age, performance status, histology, stage, status and regimens of treatment (*P* > 0.05).According to univariate analysis, the OS was significantly associated with NLR0, PLR0, NLR2, the response of 2 and 4 cycles of first line chemotherapy, status and regimens of second line treatment (*P* < 0.05), but not with stage, status of third line or beyond treatment and radiotherapy (*P* > 0.05).The multivariate analysis showed that NLR0 (*P*=0.004), the response with 4 cycles of first line chemotherapy (*P*=0.022) and status of second line treatment (*P*=0.007) were independent prognostic indicators in the 68 patients.

**Conclusion:**

The study showed that NLR0 was well connected with outcomes and NLR2 was well connected with the response to first line chemotherapy in patients with advanced non-small cell lung cancer.Therefore, NLR may be a biomarker for predicting the outcomes and response of first line chemotherapy and a potential target for management of non-small cell lung cancer.

肺癌目前是世界范围内发病率和死亡率最高的恶性肿瘤^[1]^, 其中80%为非小细胞肺癌(non-small cell lung cancer, NSCLC), 约75%的NSCLC就诊时已属中晚期^[2, 3]^, 系统性治疗仍是NSCLC重要的治疗手段。尽管目前NSCLC系统性治疗的选择较多, 如化学治疗、靶向治疗、免疫治疗等, 但整体预后仍不理想, 因此早期识别判断患者预后的指标, 对于提高患者生活质量和延长患者生存期尤为关键。中性粒细胞淋巴细胞比值(neutrophil-to-lymphocyte ratio, NLR)和血小板淋巴细胞比值(platelet-to-lymphocyte ratio, PLR)体现了机体系统性炎症的水平, 这种炎症水平与肿瘤导致的细胞破坏显著相关^[4-6]^, 多项研究表明NLR和PLR与乳腺癌^[7-9]^、胃癌^[10-12]^、结直肠癌^[13-15]^、肺癌^[16-19]^患者预后相关。近期研究表明NLR、PLR还与NSCLC免疫治疗^[20-22]^、靶向治疗^[23]^疗效和预后相关。但关于NLR、PLR动态变化与NSCLC一线化疗疗效和预后的相关研究仍较少。

本研究回顾性分析了68例2008年4月-2015年4月在北京大学第三医院接受一线化疗、符合入组标准的进展期NSCLC住院患者, 确定一线化疗前、2周期化疗后NLR和PLR的水平与患者临床病理特征、一线化疗疗效和患者预后的关系, 并分析真实世界中, 一线化疗疗效、二线治疗状态、二线治疗方案选择、三线及以上治疗状态、全程中放射治疗使用情况对患者预后的影响。

## 1 资料与方法

### 1.1 研究对象

选取2008年4月-2015年4月在北京大学第三医院接受一线化疗的进展期NSCLC初治住院患者。入组标准:组织学病理确诊为NSCLC; 有完善的影像学资料[胸腹部计算机断层扫描(computed tomography, CT)、头颅磁共振成像(magnetic resonance imaging, MRI)]可供肿瘤分期评价; 肿瘤分期包括不可手术切除的Ⅲ期和Ⅳ期患者; 一线化疗期间每2周期完善胸腹部增强CT和头颅MRI检查; 未接受过任何抗肿瘤治疗(包括放射治疗、化学治疗、生物治疗、免疫治疗、靶向治疗及中药治疗); 美国东部肿瘤协作组(Eastern Cooperative Oncology Group, ECOG)≤2分; 血常规及血生化(肝肾功能、心肌酶、电解质)等符合化疗要求; 有完善的北京大学第三医院检验科血常规检测结果可用于NLR、PLR评价, 血常规的血液采集时间在治疗前1周内; 预计生存期超过3个月; 能签署化疗知情同意并接受后续随访的。排除标准:组织学病理为非NSCLC(如小细胞肺癌); 合并两种或两种以上肿瘤; 合并血液系统疾病; 合并免疫系统疾病; 合并乙型肝炎或丙型肝炎病毒感染; 既往长期接受激素药物治疗; 治疗前分期评价不足; 治疗期间未进行影像学采集可供疗效评价; 一线化疗期间出现不可逆重度骨髓抑制; 一线化疗间隔推迟1个月以上; 缺乏北京大学第三医院检验科血常规检测用于评价NLR、PLR; 评价NLR、PLR, 采集血常规前有激素应用史或合并感染性发热; 评价NLR、PLR时, 血常规提示合并2度及以上骨髓抑制; 不能接受后续规律随访。纳入符合条件的患者68例。

### 1.2 临床资料收集

采集并记录患者确诊时的临床资料, 包括年龄、性别、肿瘤分期、病理类型、*EGFR*基因突变检测情况等; 患者肿瘤治疗资料, 包括一线化疗方案、一线治疗期间2周期、4周期后疗效评价、后续治疗情况(如二线、三线及以上的治疗方案, 包括放射治疗、靶向治疗、化学治疗等)、治疗期间的影像学资料; 一线化疗期间第1周期和第3周期前1周内血常规中的中性粒细胞、淋巴细胞和血小板计数。

### 1.3 评价标准

肿瘤分期依据国际肺癌研究协会颁布的第7版分期标准^[24]^; 疗效评价指标包括近期和远期疗效。近期疗效依照实体瘤疗效评价标准1.1版^[25]^分为完全缓解(complete response, CR)、部分缓解(partial response, PR)、疾病稳定(stable disease, SD)和疾病进展(progressive disease, PD), 获得CR或PR的患者4周或以后确认。远期疗效本研究仅对总生存期(overall survival, OS)进行观察。OS定义为从初次治疗开始至死亡或随访终点时间; NLR和PLR分别是中性粒细胞计数与淋巴细胞计数的比值和血小板计数与淋巴细胞计数的比值。

### 1.4 观察指标

NLR、PLR、一线化疗的近期疗效和OS; 主要记录一线化疗前和2周期化疗后NLR和PLR, 分别记录为基线NLR0、PLR0和2周期后的NLR2和PLR2;一线化疗近期疗效主要记录2周期和4周期的近期疗效, 其中4周期疗效中的PD包括2周期评价为PD的出组患者。

### 1.5 随访

通过定期来院或电话随访, 随访开始时间为2008年4月, 末次随访时间为2018年3月31日, 随访率100.0%, 最短随访时间38 d, 最长2, 137 d。

### 1.6 统计学方法

应用SPSS 19.0统计学软件分析。率的比较采用卡方检验或*Fisher*精确检验; 相关性分析应用*Pearson*检验; 绘制NLR、PLR的受试者工作特征曲线(receiver operating characteristic curve, ROC), 应用曲线下面积(area under the curve, AUC)来评价其诊断价值; 应用*Kaplan-Meier*方法进行生存分析, *Log-rank*检验差异性; 多因素分析采用*Cox*多因素分析模型, 逐步后退法(backward, walds)。全部统计检验均为双侧概率检验, 检验水准α=0.05, 以*P* < 0.05为差异有统计学意义。

## 2 结果

### 2.1 患者一般临床特征

共68例患者入组, 男50例, 女18例; 中位年龄为60.5岁(37岁-81岁), ≥70岁者11例(16.2%), < 70岁者57例(83.8%); ECOG 0/1分者居多, 共66例(97.1%); Ⅳ期患者46例(67.6%)最多, Ⅲb期15例(22.1%), Ⅲa期7例(10.3%); 68例NSCLC患者中, 腺癌42例(61.8%)最多, 鳞癌24例(35.3%)次之, 腺鳞癌2例(2.9%); 本组患者中45例接受了*EGFR*基因检测, 8例含有*EGFR*突变, 37例为EGFR野生型; 一线化疗中接受紫杉类药物为主联合治疗的18例(26.5%), 吉西他滨为主的26例(38.2%), 培美曲塞为主的23例(33.8%); 一线化疗后未见CR患者, 其中2周期化疗后PR 13例(19.1%), SD 45例(66.2%), PD 10例(14.7%), 4周期化疗后PR 19例(27.9%), SD 33例(48.5%), PD 16例(23.5%); 68例患者中, 52例(76.5%)接受了二线治疗, 单纯化疗21例(30.9%), 放疗联合化疗20例(29.4%), 靶向治疗11例(16.2%); 接受三线及以上治疗的患者共25例(36.8%), 见表 1。

**1:** 68例进展期非小细胞肺癌患者的临床特征 The clinical features of 68 cases with advanced non-small cell lung cancer

Clinical features	Cases (*n*)	%
Age (yr)		
≥70	11	16.2
< 70	57	83.8
Range	37-81	
Median age	60.5	
Gender		
Female	18	26.5
Male	50	73.5
Performance status (ECOG)		
0	17	25.0
1	49	72.1
2	2	2.9
Stage		
Ⅲa	7	10.3
Ⅲb	15	22.1
Ⅳ	46	67.6
Type of histology		
Adenocarcinoma	42	61.8
Squamous cell carcinoma	24	35.3
Adenosquamous carcinoma	2	2.9
Status of *EGFR* gene		
EGFR wide-type	37	54.4
*EGFR* mutation	8	11.8
EGFR-unknown	23	33.8
First line chemotherapeutic regimen		
Taxanes-based	18	26.5
Gemcitabine-based	26	38.2
Pemtrexed-based	23	33.8
Others	1	1.5
Response after 2 cylces of chemotherapy		
PR	13	19.1
SD	45	66.2
PD	10	14.7
Response after 4 cycles of chemotherapy		
PR	19	27.9
SD	33	48.5
PD	16	23.5
Second line treatment		
No	16	23.5
Yes	52	76.5
Chemotherapy (CT)	21	30.9
Radiotherapy (RT) combined with CT	20	29.4
Targeted therapy	11	16.2
Third line or beyond treatment		
Yes	25	36.8
No	43	63.2
CT:chemotherapy; RT:radiotherapy.		

### 2.2 NLR、PLR及其动态变化与患者临床特征之间的相关性

本研究中NLR0范围为0.81-11.39, NLR2范围为0.39-11.43;一线化疗后, NLR2较NLR0显著下降, 分别为(2.69±2.06)和(3.94±2.12)(*P*=0.000);PLR0范围为68.50-413.89, PLR2范围为56.67-720.0, PLR2同PLR0相比无显著变化, 分别为(188.31±115.75)和(177.49±81.20)(*P*=0.395)。*Pearson*相关分析表明NLR0(*r*=-0.332, *P*=0.006)、PLR0(*r*=-0.346, *P*=0.004)、NLR2(*r*=-0.260, *P*=0.032)与OS呈负相关, PLR2与OS(*r*=-0.238, *P*=0.050)有相关趋势。NLR0、PLR0、PLR2与患者性别、年龄、ECOG评分、病理类型、EGFR检测结果、肿瘤分期、一线化疗方案、2周期化疗疗效、4周期化疗疗效、二线治疗状态、二线治疗方案和三线及以上治疗状态均无关(*P* > 0.05)。NLR2除与ECOG评分(*r*=0.241, *P*=0.048)相关外, 与其他临床特征无关(*P* > 0.05)。

### 2.3 NLR、PLR的ROC曲线及界值

确定68例患者生存情况包括死亡和存活两组, NLR、PLR的ROC曲线见图 1。图 1A是NLR0和PLR0对患者生存的预测能力, NLR0曲线下面积(area under the curve, AUC)为0.756(95%CI:0.545-0.968, *P*=0.027), 最佳判读值2.155, 灵敏度为86.9%, 特异度为71.4%;PLR0的AUC为0.742(95%CI:0.554-0.931, *P*=0.037), 最佳判读值129.265, 灵敏度为67.2%, 特异度为71.4%;图 1B是NLR2和PLR2对患者生存的预测能力, NLR2的AUC为0.776(95%CI:0.605-0.947, *P*=0.017), 最佳判读值1.65, 其预测灵敏度为73.8%, 特异度为71.4%。PLR2的AUC为0.637(95%CI:0.382-0.892, *P*=0.238)。故本研究定义NLR0、PLR0和NLR2高的界值分别为≥2.16、≥130.0和≥1.65。

**1:**
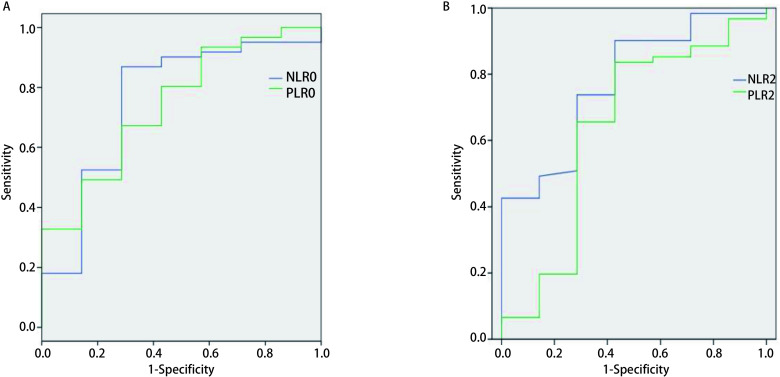
NLR0、PLR0、NLR2及PLR2预测68例NSCLC患者预后的ROC曲线。A:NLR0和PLR0预测患者预后的ROC曲线; B:NLR2和PLR2预测患者预后的ROC曲线。 Receiver operating characteristic(ROC) curve of NLR0, PLR0, NLR2 and PLR2 in predicting prognosis of 68 cases with non-small cell lung cancer.A:ROC curve of NLR0 and PLR0 in predicting the prognosis; B:ROC curve of NLR2 and PLR2 in predicting the prognosis.

### 2.4 高NLR、PLR与患者临床特征间的关系

本研究中, 治疗前高NLR0、PLR0在患者年龄、性别、ECOG评分、分期、病理类型、一线治疗方案、2周期化疗疗效以及4周期化疗疗效、二线治疗状态及方案选择、三线及以上治疗状态等组内无显著差异(*P* > 0.05), 见表 2和表 3; 而2周期化疗后, 高NLR2在2周期化疗后PR和SD组中的比例分别为53.8%(7/13)和66.7%(30/45), 显著低于PD组中的100%(10/10)(*P*=0.040), 在4周期化疗后PR和SD组中的比例分别为52.6%(10/19)和63.6%(21/33), 显著低于PD组中的100%(16/16)(*P*=0.006), 但在患者年龄、性别、ECOG评分、分期、病理类型、一线治疗方案、二线治疗与否及类型、三线及以上治疗与否等组内无显著差异(*P* > 0.05), 见表 2。

**2:** 高NLR同68例进展期非小细胞肺癌患者临床特征间的关系 The relationship between high NLR and clinical features of 68 cases with advanced non-small cell lung cancer

Clinical features	*n*	High-NLR0^*^ [*n* (%)]	χ^2^	*P*	High NLR2^**^ [*n* (%)]	χ^2^	*P*
Age (yr)			3.102	0.107		0.992	0.482
≥70	11	11 (100.0)			9 (81.8)		
< 70	57	44 (77.2)			38 (66.7)		
Gender			0.153	0.733		0.069	1.000
Female	18	14 (77.8)			12 (66.7)		
Male	50	41 (82.0)			35 (70.0)		
Performance status (ECOG)			4.090	0.106		3.412	0.188
0	17	11 (64.7)			9 (52.9)		
1	49	42 (85.7)			36 (73.5)		
2	2	2 (100.0)			2 (100.0)		
Stage			2.964	0.234		0.750	0.723
Ⅲa	7	4 (57.1)			5 (71.4)		
Ⅲb	15	13 (86.7)			9 (60.0)		
Ⅳ	46	38 (82.6)			33 (74.7)		
Type of histology			1.173	0.689		4.977	0.092
Adenocarcinoma	42	35 (83.3)			25 (59.5)		
Squamous cell carcinoma	24	18 (75.0)			20 (83.3)		
Adenosquamous carcinoma	2	2 (100.0)			2 (100.0)		
Status of *EGFR* gene			0.224	1.000		2.312	0.315
EGFR wide-type	37	30 (81.1)			25 (67.6)		
*EGFR* mutation	8	6 (82.6)			4 (50.0)		
EGFR-unknown	23	19 (82.6)			18 (78.3)		
First line chemotherapeutic regimen			1.263	0.744		1.973	0.640
Taxanes-based	18	15 (83.3)			12 (66.7)		
Gemcitabine-based	26	22 (84.6)			20 (76.9)		
Pemtrexed-based	23	17 (73.9)			14 (60.9)		
Others	1	1 (100.0)			1 (100.0)		
Response after 2 cylces of chemotherapy			2.461	0.339		6.015	0.040
PR	13	12 (92.3)			7 (53.8)		
SD	45	34 (75.6)			30 (66.7)		
PD	10	9 (90.0)			10 (100.0)		
Response after 4 cycles of chemotherapy			1.150	0.572		10.033	0.006
PR	19	16 (84.2)			10 (52.6)		
SD	33	25 (75.8)			21 (63.6)		
PD	16	14 (87.5)			16 (100.0)		
Second line treatment			0.593	0.504		0.339	0.759
No	16	14 (87.5)			12 (75.0)		
Yes	52	41 (78.8)			35 (67.3)		
CT	21	18 (85.7)	1.691	0.688	18 (85.7)	5.755	0.128
RT combined with CT	20	15 (75.0)			13 (65.0)		
Targeted therapy	11	8 (72.7)			5 (45.5)		
Third line or beyond treatment			3.160	0.111		0.154	0.789
Yes	25	23 (92.0)			18 (72.0)		
No	43	32 (74.4)			29 (67.4)		
CT:chemotherapy; RT:radiotherapy; NLR:neutrophil to lymphocyte ratio; PLR:platelet to lymphocyte ratio; NLR0 refers to the baseline NLR without any treatment and NLR2 refers to the NLR after 2 cycles chemotherapy; ^*^High NLR0 refers to score of NLR≥2.16;^**^High NLR2 refers to score of NLR≥1.65.The score was the value of neutrophil to lymphocyte ratio.							

**3:** 高PLR同68例进展期非小细胞肺癌患者临床特征间的关系 The relationship between high PLR and clinical features of 68 cases with advanced non-small cell lung cancer

Clinical features	Cases (*n*)	High PLR0^***^ [*n* (%)]	χ^2^	*P*
Age (yr)			0.001	1.000
≥70	11	7 (63.6)		
< 70	57	36 (63.2)		
Gender			0.850	0.407
Female	18	13 (72.2)		
Male	50	30 (60.0)		
Performance Status (ECOG)			2.028	0.357
0	17	9 (52.9)		
1	49	32 (65.3)		
2	2	2 (100.0)		
Stage			1.660	0.499
Ⅲa	7	3 (42.9)		
Ⅲb	15	9 (60.0)		
Ⅳ	46	31 (67.4)		
Type of histology			1.200	0.665
Adenocarcinoma	42	26 (61.9)		
Squamous cell carcinoma	24	15 (62.5)		
Adenosquamous carcinoma	2	2 (100.0)		
Status of *EGFR* gene			1.798	0.478
EGFR wide-type	37	21 (56.8)		
*EGFR* mutation	8	5 (62.5)		
EGFR-unknown	23	17 (73.9)		
First line chemotherapeutic regimen			2.618	0.477
Taxanes-based	18	10 (55.6)		
Gemcitabine-based	26	18 (69.2)		
Pemtrexed-based	23	15 (65.2)		
Others	1	0 (0.0)		
Response after 2 cylces chemotherapy			0.266	0.870
PR	13	9 (69.2)		
SD	45	28 (62.2)		
PD	10	6 (60.0)		
Response after 4 cycles chemotherapy			0.445	0.807
PR	19	11 (57.9)		
SD	33	21 (63.6)		
PD	16	11 (68.8)		
Second line treatment			0.274	0.769
No	16	11 (68.8)		
Yes	52	32 (61.5)		
CT	21	14 (66.7)	2.249	0.522
RT combined with CT	20	10 (50.0)		
Targeted therapy	11	8 (72.7)		
Third line or beyond treatment			1.306	0.304
Yes	25	18 (72.0)		
No	43	25 (58.1)		
CT:chemotherapy; RT:radiotherapy; PLR0 refers to the baseline PLR without any treatment.^***^High PLR0 refers to score of PLR≥130 and the score was the value of platelet to lymphocyte ratio.				

### 2.5 生存分析

*Pearson*相关分析表明68例患者OS与NLR0(*r*=-0.332, *P*=0.006)、PLR0(*r*=-0.346, *P*=0.004)、NLR2(*r*=-0.260, *P*=0.032)、4周期化疗疗效(*r*=-0.349, *P*=0.004)、二线治疗状态(*r*=0.343, *P*=0.004)、二线治疗选择(*r*=0.495, *P*=0.000)显著相关, 但与患者性别、年龄、ECOG、肿瘤分期、病理类型、EGFR检测结果、一线化疗方案、2周期化疗疗效、三线及以上治疗状态无关(*P* > 0.05)。*Kaplan-Meier*生存分析表明:①低NLR0组中位OS 1, 372.0 d (95% CI:146.7-2, 597.3)显著高于高NLR0组的430.0 d(95%CI:329.3-530.7)(*P*=0.001), 见表 4和图 2A; ②低NLR2中位OS 907.0 d(95%CI:434.1-1, 379.9)显著高于高NLR2组的369.0 d(95%CI:252.1-485.9)(*P*=0.001), 见表 4和图 2B; ③低PLR组的中位OS 675.0 d(95%CI:355.1-994.9)显著高于高PLR组的393.0 d (95%CI:296.6-489.4 d)(*P*=0.030), 见表 4和图 2C; ④接受二线治疗组的中位OS 561.0 d(95%CI:397.3-724.7 d)显著高于未接受二线治疗组的268.0 d(95%CI:166.1-369.9)(*P*=0.000), 见表 4和图 2E。在二线治疗中, 靶向治疗组中位OS 1, 313.0 d(95%CI:786.8-1, 839.2)高于化疗组466.0 d(95%CI:346.4-585.6)和化疗联合放疗组443.0 d(95%CI:54.4-831.6), 均显著高于未治疗组268.0 d(95%CI:166.1-369.9)(*P*=0.000), 见表 4和图 2F; ⑤2周期化疗后获得PR和SD组的中位OS分别为561.0 d(95%CI:96.0-1, 026.0)和497.0 d(95%CI:293.3-700.7), 显著高于PD组的233.0 d(95%CI:101.3-364.7)(*P*=0.003), 见表 4和图 2G; 4周期化疗后获得PR和SD组的中位OS分别为719.0 d(95%CI:393.4-1, 044.6)和497.0 d(95%CI:291.6-702.4), 显著高于PD组的312.0 d(95%CI:192.4-431.6)(*P*=0.000), 见表 4和图 2H; ⑥本研究中, 患者的中位OS在肿瘤分期、接受三线及以上治疗与否和肿瘤患者全程是否接受放疗的组内未见显著性差异(*P* > 0.05), 见表 4和图 2D、图 2I和图 2J。

**4:** 影响68例进展期非小细胞肺癌患者生存的单因素分析 Univariate analysis of overall survival in 68 cases with advanced non-small cell lung cancer

Variabe	*n*	Median OS (d)	95%CI		*P* value
			Lower	Upper	
NLR0					0.001
< 2.16	13	1, 372.0	146.7	2, 597.3	
≥2.16	55	430.0	329.3	530.7	
NLR2					0.002
< 1.65	21	907.0	434.1	1, 379.9	
≥1.65	47	369.0	252.1	485.9	
PLR0					0.030
< 130	25	675.0	355.1	994.9	
≥130	43	393.0	296.6	489.4	
Stage					0.243
Ⅲa	7	1, 297.0	152.4	2, 441.6	
Ⅲb	15	382.0	305.0	459.0	
Ⅳ	46	458.0	342.8	573.2	
Second-line treatment					0.000
Yes	52	561.0	397.3	724.7	
No	16	268.0	166.1	369.9	
Type of second line treatment					0.000
No	16	268.0	166.1	369.9	
CT	21	466.0	346.4	585.6	
RT+CT	20	443.0	54.4	831.6	
Targeted therapy	11	1, 313.0	786.8	1, 839.2	
Response after 2 cycles of chemotherapy					0.003
PR	13	561.0	96.0	1, 026.0	
SD	45	497.0	293.3	700.7	
PD	10	233.0	101.3	364.7	
Response after 4 cycles of chemotherapy					0.000
PR	19	719.0	393.4	1, 044.6	
SD	33	497.0	291.6	702.4	
PD	16	312.0	192.4	431.6	
Third line treatment					0.259
Yes	25	695.0	497.5	892.5	
No	43	350.0	254.9	445.1	
Radiotherapy					0.286
Yes	25	635.0	302.6	967.4	
No	43	458.0	311.5	604.5	

**2:**
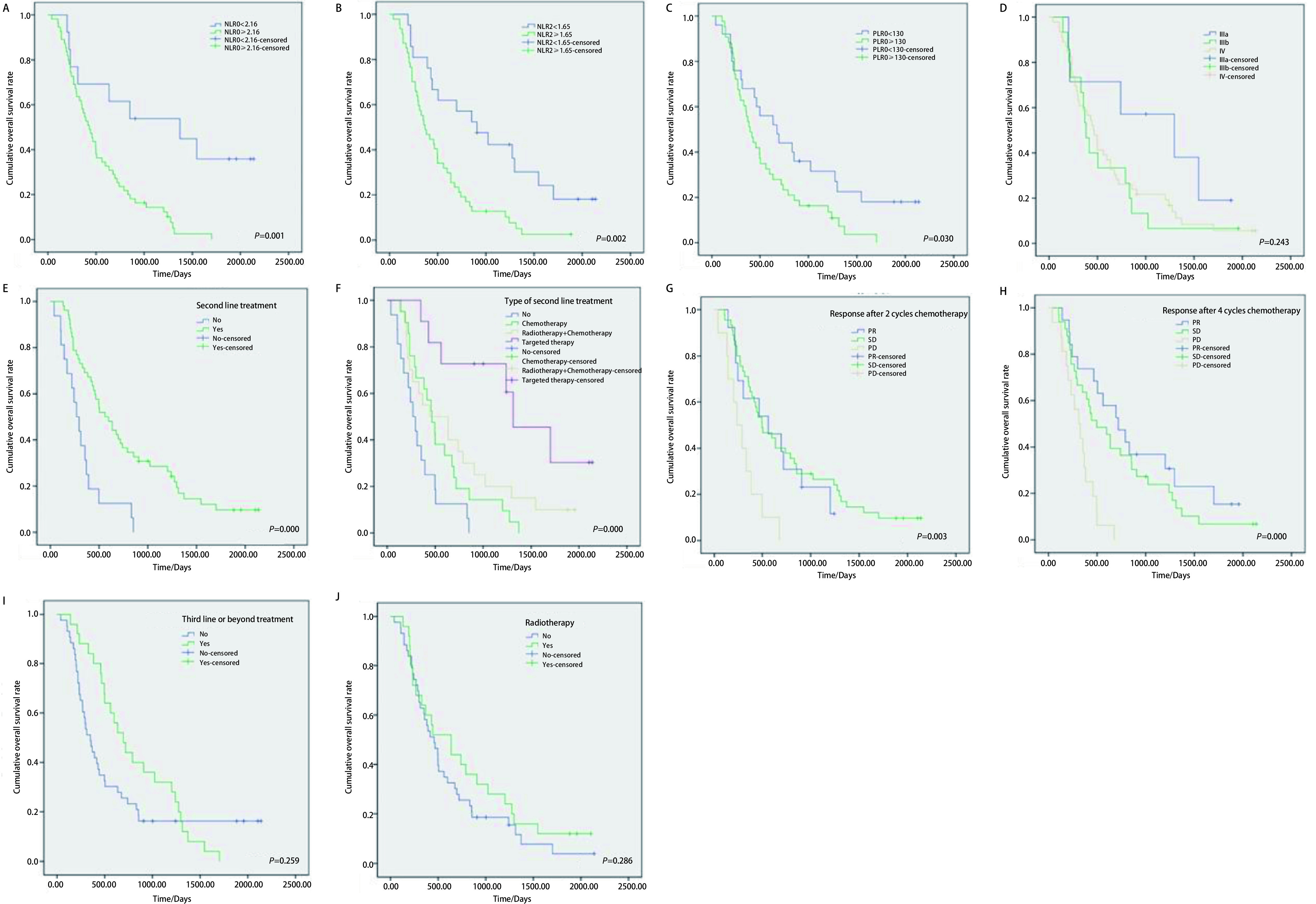
*Kaplan-Meier*生存曲线分析。A:NLR0分组对OS的影响; B:NLR2分组对OS的影响; C:PLR0分组对OS的影响; D:分期对OS的影响; E:二线治疗对OS的影响; F:二线治疗类型对OS的影响; G:一线治疗2周期患者缓解情况对OS的影响; H:一线治疗4周期患者缓解情况对OS的影响; I:三线及以上治疗与否对OS的影响; J:全程接受放疗与否对生存期的影响。 *Kaplan-Meier* cumulative overall survival curves analysis.A:The OS curves of different groups about NLR0;B:The OS curves of different groups about NLR2;C:The OS curves of different groups about PLR0;D:The OS curves of different groups about stage; E:The OS curves of different groups about second line treatment; F:The OS curves of different treatment methods in second line treatment; G:The OS curves of different groups about the response after first line treatment with 2 cycles of chemotherapy; H:The OS curves of different groups about the response after first line treatment with 4 cycles of chemotherapy; I:The OS curves of different groups about third line or beyond treatment; J:The OS curves of different groups about radiotherapy during the treatment.

### 2.6 *Cox*回归分析

在校对患者NLR0、NLR2、PLR0、2周期疗效、4周期疗效、二线治疗状态和二线治疗方案等因素后, 多因素分析表明NLR0(*P*=0.004)、4周期一线化疗疗效(*P*=0.022)和二线治疗方案(*P*=0.007)是OS的独立预测因素, 而NLR2、PLR0、2周期一线化疗疗效、二线治疗状态对OS无显著预测价值(*P* > 0.05), 见表 5。

**5:** 影响68例非小细胞肺癌患者预后的多因素分析 *Cox* regression analysis of the overall survival in 68 cases with non-small cell lung cancer

Characteristic	Regression coefficient *β*	Standard error	Wald	*P* value	Exp(B)	95%CI
Overall survival						
NLR0	1.328	0.465	8.155	0.004	3.772	1.516-9.382
NLR2	0.447	0.352	1.611	0.204	1.563	0.784-3.115
PLR0	0.070	0.351	0.040	0.841	1.073	0.540-2.133
Response after 2 cycles CT	-0.126	0.373	0.114	0.736	0.882	0.425-1.831
Response after 4 cycles CT	0.593	0.259	5.234	0.022	1.810	1.089-3.008
Second line treatment	-0.380	0.402	0.895	0.344	0.684	0.540-2.133
Type of second line treatment	-0.514	0.190	7.291	0.007	0.598	0.412-0.869

## 3 讨论

近年来, 随着NSCLC驱动基因和免疫检测点的识别、新药的不断出现、多学科综合诊疗的合理实施, 使得NSCLC的预后得到了显著改善, 但NSCLC整体5年生存率仍非常低, 仅为18%^[1, 3]^。人们一直在探寻和NSCLC预后相关的生物标志, 拟通过积极干预获得更好的治疗效果。NLR和PLR是机体系统性炎症的体现, 作为血液学评价方法既方便又经济, 目前已在胃癌^[11, 12, 26]^、结直肠癌^[13, 14]^、胆囊癌及胆管癌^[27]^、乳腺癌^[7, 8]^、卵巢癌^[28]^和NSCLC^[16, 18, 21]^等多种肿瘤中证实NLR或PLR与患者预后显著相关, 而NLR、PLR动态变化与患者治疗疗效和预后的关系也成为目前研究热点, 但相关报道较少。

本研究中NLR0(*P*=0.006)、PLR0(*P*=0.004)和NLR2(*P*=0.032)同患者OS呈负相关, 但PLR2与OS无关。低NLR0组中位OS 1, 372.0 d显著高于高NLR0组的430.0 d(*P*=0.001);低NLR2组中位OS 907.0 d显著高于高NLR2组的369.0 d(*P*=0.001);低PLR0组的中位OS 675.0 d显著高于高PLR0组的393.0 d。多因素分析表明NLR0是OS的独立预测因素之一(HR=3.772, 95%CI:1.516-9.382, *P*=0.004)。与Cedres等^[16]^在171例晚期NSCLC患者中的早期报道类似:低NLR组与高NLR组的中位OS分别为11.1个月和5.6个月(*P*=0.017)。Peng等^[29]^和Sacdalan等^[22]^两项荟萃分析也表明NSCLC患者中高NLR组的预后更差(*P* < 0.05)。Liu等^[19]^在934例肺癌患者中证实高NLR组的预后差。此外, Lin等^[23]^在81例*EGFR*突变接受靶向药物一线治疗的NSCLC患者中, 也发现高NLR组患者预后差。可见, 不论是否存在基因突变, NLR与患者预后显著相关。但本研究中低NLR0组生存期1, 372.0 d较Cedres等^[16]^报道的11.1个月显著延长, 其差异产生原因:①本组研究中患者后续二线治疗患者较多, 部分患者应用了EGFR靶向治疗; ②两组NLR确定的界值不同, 本组研究中高NLR≥2.16, Cedres研究中高NLR≥5。

本研究中NLR在化疗后有明显下降, NLR2和NLR0分别为(2.69±2.06)和(3.94±2.12)(*P*=0.000);PLR在化疗后无显著变化(*P*=0.395)。NLR2的水平与化疗疗效显著相关, 高NLR2在2周期化疗后PR和SD组中的比例分别为53.8%(7/13)和66.7%(30/45), 显著低于PD组中的100%(10/10)(*P*=0.040), 在4周期化疗后PR和SD组中的比例分别为52.6%(10/19)和63.6%(21/33), 显著低于PD组中的100%(16/16)(*P*=0.006), 但未发现NLR0、PLR0与疗效相关。Liu等^[19]^近期研究发现化疗前后PLR无显著变化, 但NLR在化疗后有上升趋势, CR患者组中的NLR水平显著低于PR+SD+PD组。本研究结果与Liu等^[19]^产生差异的原因:①入组患者病理类型和分期不同, Liu等^[19]^研究中含有153例小细胞肺癌患者, 且含有早期肺癌患者; ②肿瘤化疗方案或许不同; ③化疗缓解率不同, 本研究中无CR患者, Liu等^[19]^报道中CR率高达23%, 或许与其研究中小细胞肺癌样本量较大有关。本研究中高NLR2组疗效差, 与Yao等^[18]^在182例肺癌患者中发现基线高NLR组铂类药物一线化疗疗效差、Peng等^[29]^荟萃分析表明高NLR与疗效呈反比以及Botta等^[30]^报道高NLR组NSCLC患者贝伐单抗疗效更差的结果相一致, 但本研究并未发现基线NLR与化疗疗效相关, 其原因考虑:①四组研究NLR组界值不同; ②考虑NLR为体内炎症体现, 或许其动态变化更能反应治疗效果。这也与近期NLR在免疫治疗中的报道相一致:Nakya等^[21]^发现治疗4周后NLR < 3是nivolumab治疗NSLCL疗效的预测标志; Suh等^[31]^治疗6周后发现NLR < 5是抗PD-1抗体治疗NSCLC疗效更好的标志。此外, Kiriu等^[20]^在19例接受nivolumab治疗的NSCLC患者中发现, PD患者(5/7)中NLR显著升高, 且在第1周期、第2周期治疗后, NLR升高超过30%患者的至疾病治疗失败时间较NLR稳定或下降的患者显著缩短。本研究中NLR动态变化在NSCLC中的作用与Chua等^[14]^在结直肠癌中的发现类似, Chua等在162例接受姑息性化疗的晚期结直肠癌患者中, 发现治疗前NLR > 5的患者, 在治疗1周期后下降至NLR < 5的患者PFS显著高于持续NLR > 5的患者。因此, NLR动态变化与肿瘤化疗疗效相关。

NLR和PLR影响患者预后和疗效的机制仍不清楚, NLR、PLR被认为是肿瘤微环境中炎症变化的反映^[32]^, 中性粒细胞和血小板在体内可以促进肿瘤血管形成、肿瘤细胞增殖和转移^[5, 6, 33]^, 从而促进肿瘤进展。而淋巴细胞在肿瘤免疫监视中发挥着重要作用, 其可以抑制肿瘤的进展^[4]^。因此, 外周血中NLR和PLR的失衡为理解肿瘤进展和患者预后提供了新的诊疗视角。但关于NLR、PLR对肿瘤预后和治疗疗效的影响仍有许多问题亟待解决:①NLR和PLR界值统一; ②NLR和PLR动态变化对治疗疗效影响; ③通过干预NLR和PLR能否改善患者生存等, 这些均有待于大样本前瞻性临床研究来进行解答。

本研究生存分析发现一线化疗后2周期疗效、4周期疗效、二线治疗状态、二线治疗方案选择均与患者OS显著相关(*P* < 0.05), 多因素分析表明4周期化疗疗效和二线治疗状态也是本组患者预后的独立预测因素。4周期化疗后PR和SD组的中位OS分别为719.0 d和497.0 d, 显著高于PD组的312.0 d(*P*=0.000);接受二线治疗组的中位OS 561.0 d显著高于未接受二线治疗组的268.0 d(*P*=0.000), 靶向治疗组中位OS 1, 313.0 d显著高于化疗的466.0 d、化疗联合放疗的443.0 d, 均显著高于未治疗的268.0(*P*=0.000)。本研究结果与Sirohi等^[34]^在320例回顾分析NSCLC患者中的发现相一致, 即2周期化疗后PR患者2年生存率为23%, 显著高于疗效稳定组11%(*P*=0.002)。4周期疗效对预后的影响与Ross等^[35]^荟萃分析1, 139例NSCLC患者、Liu等^[36]^224例NSCLC患者中分析结果相一致, 4周期与6周期或以上相比, 对生存影响无显著性差异。但本研究中并未发现患者的中位OS在肿瘤分期、接受三线及以上治疗与否和肿瘤患者全程是否接受放疗的组内有显著性差异, 或许与本研究样本量偏小有关。

本研究的局限性在于样本量偏小, 存在选择性偏倚可能。但本研究结果进一步证实了NLR和PLR在NSCLC中对预后的影响, 高NLR0、高PLR0组患者的OS更差, NLR的动态变化与患者的疗效显著相关, 2周期、4周期化疗后PD组中高NLR2比例高达100.0%。多因素分析表明NLR0、一线化疗4周期疗效、二线治疗状态是患者OS的独立预测因素。综上可见, NLR0是预测进展期NSCLC预后的理想指标, 也是NSCLC治疗潜在干预的靶点, 需要进一步大样本量的前瞻性临床研究进行验证。
